# Improvement of Adherence with Occlu-Pad Therapy for Pediatric Patients with Amblyopia

**DOI:** 10.1155/2018/2394562

**Published:** 2018-11-22

**Authors:** Satoru Totsuka, Tomoya Handa, Hitoshi Ishikawa, Nobuyuki Shoji

**Affiliations:** ^1^Department of Ophthalmology, Kitasato University, 1-15-1 Kitasato, Sagamihara 252-0375, Japan; ^2^Department of Rehabilitation, Orthoptics of Visual Science, School of Allied Health Sciences, Kitasato University, 1-15-1 Kitasato, Sagamihara 252-0373, Japan

## Abstract

We aimed to examine visual acuity improvement effect and adherence in amblyopia training using tablet type vision training equipment (Occlu-pad). The subjects were 138 patients with amblyopia (average age of 5.5 ± 1.6 years old); their amblyopic visual acuity at the start of training was logMAR 0.15 to 1.3. Occlu-pad is a device that processes images such that amblyopic eyes can only view the image as it passes through polarized glasses; this is achieved by peeling off the polarizing film layer in the liquid crystal display of an iPad (Apple). Amblyopia training comprised either the instructional training with Occlu-pad or the eye patch (Patching) as a family training, after wearing perfectly corrected glasses. Visual acuity improvement following amblyopia training by Occlu-pad and Patching was significantly different after 6 months in patients with anisometropic amblyopia (p <0.05). In patients with strabismic amblyopia, a significant difference between training methods was observed after 9 months (p <0.05). Use of the Occlu-pad resulted in better adherence for patients with either anisometropic amblyopia or strabismic amblyopia; a significant difference in adherence was observed after 3 months, compared with Patching (p <0.05). Amblyopia training with Occlu-pad supports greater visual acuity improvement and adherence than Patching.

## 1. Introduction

Amblyopia is reportedly present in approximately 5% of all children [1]. If it is not discovered and corrected during the visual susceptibility period, poor lifetime visual acuity can occur, ultimately causing visual impairment among elderly people. Epidemiologic studies [2] have shown that the rates of falls and blindness in both eyes are twofold higher in amblyopic patients than in people without amblyopia. Treatment of amblyopia is therefore extremely important.

In conventional clinical ophthalmology, the gold standard for amblyopia treatment is occlusion therapy (patching), which involves the use of an eyepatch on the healthy eye [3–5]. Patching can improve visual acuity in patients with amblyopia. However, 15% to 50% of affected patients may not achieve normal visual acuity, despite a lengthy course of treatment [6]. Moreover, adherence is often problematic. Enhancement of adherence is an important aspect that determines the success or failure of amblyopia treatment. Indeed, Patching training time adherence falls to 20% of the prescribed duration after 3 months [7]. Therefore, separate explanations have been provided to parents and children, using pamphlets and calendars [8], in attempts to increase the frequency of hospital visits. They understand the importance of the treatment and the benefit of attending regularly and are therefore more likely to comply [7]. However, adherence to the prescribed duration of training time depends on commitments from parents, and it has been difficult to measure true adherence.

Recently, we developed an amblyopia treatment device within a tablet that is expected to show great efficacy [9]; in Japan, the device is known as “Occlu-pad” (outside Japan, it is known as “Occlu-tab”). Occlu-pad has a key function in that the actual training time can be recorded automatically, and accurate evaluation of adherence is possible. In this study, we examined the usefulness of amblyopia training with Occlu-pad and assessed patient adherence to amblyopia training time.

## 2. Materials and Methods

This research conformed to the tenets of the Declaration of Helsinki and was approved by the Kitasato University School of Medicine and Hospital Ethics Committee (Approval No. B14-65).

Among 138 patients with amblyopia in this study, 72 underwent Occlu-pad training (Occlu-pad group: anisometropic amblyopia, 35 patients; strabismic amblyopia, 37 patients); 66 underwent patching training (Patching group: anisometropic amblyopia, 35 patients; strabismic amblyopia, 31 patients). Patient characteristics in the Occlu-pad and Patching groups before training are shown in Table 1. There were no statistically significant differences (p<0.05; Scheffe's test) in age or visual acuity at the start of training.

**Figure 1:**
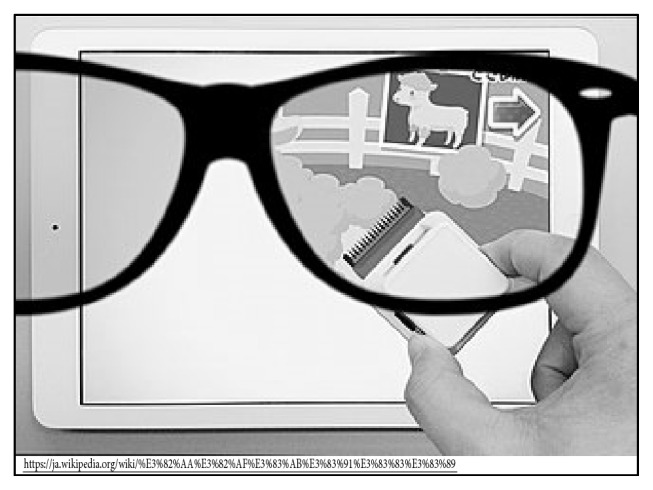
Occlu-pad [15].

Occlu-pad is a device that processes images such that only eyes viewing through polarized glasses (amblyopic eyes) can see the images; this is achieved by peeling off the polarizing film layer (white screen technology) in the liquid crystal display of an iPad (Apple) (Figure 1). Patients are trained by wearing dedicated polarized glasses and manipulating the application in Occlu-pad. Polarized glasses appear to contain the same material in both left and right lenses; notably, a polarizing film is worn on the lens of the amblyopic eye, whereas a light-shielding film (with color tone adjusted to be equal to that of the polarizing film) is worn on the lens of the fellow eye. As a result, images can be presented solely to amblyopic eyes wearing polarized glasses, and it is possible to perform training of amblyopic eyes without experiencing the discomfort associated with keeping both eyes open.

For the Occlu-pad group, refraction examination was performed via controlled palsy with atropine or cyclopentolate; the Occlu-pad group wore full correction glasses. Occlu-pad training content was lent to the patients, who were instructed to use the application 1 hour per day. During training with the Occlu-pad, patients played a game requiring hand-eye coordination; training results were automatically saved in the Occlu-pad. Orthoptists could then confirm whether the child was training properly; to do so, they retrieved the Occlu-pad at each visit to extract actual adherence data.

For the Patching group, refraction examination was also performed via controlled palsy with atropine or cipropride; the Patching group wore full correction glasses. Training comprised Patching for 3 hours; this involved occlusion of the fellow eye (eye patch®, Kawamoto Sangyo). The results of family training were listed on the calendar by the parents of the children, and adherence to the prescribed training time was confirmed.

Wearing full correction glasses, distance-corrected vision was measured at an angular visual acuity table (Handaya Co., Ltd.). All patients visited the hospital each month; visual acuity improvement effects were recorded for 12 months. Adherence was determined with the following calculation equation:

(1) Adherence%=Enforcement  time×number  of  daysinstruction  time  total  days×100

Distance vision was converted to logMAR and averaged. Visual acuity improvement was evaluated at 3, 6, 9, and 12 months and was compared with baseline visual acuity (at the start of training). One-way ANOVA was used to compare visual acuity improvement between Occlu-pad and Patching. Adherence was compared using the Mann-Whitney U test.

## 3. Results and Discussion

### 3.1. Results

**Figure 2:**
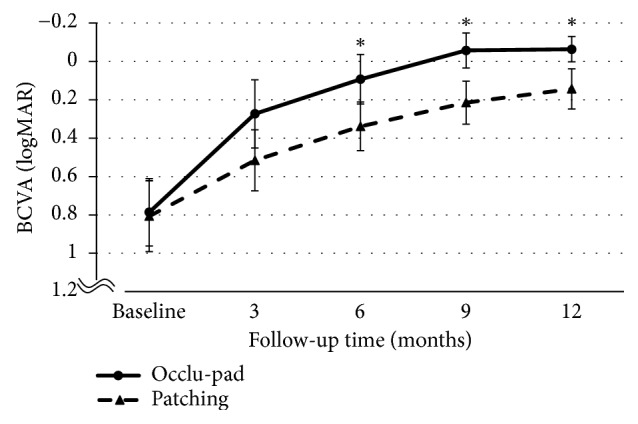
Best-corrected visual acuity (BCVA) for anisometropic amblyopia patients, from baseline to 3, 6, 9, and 12 months. Occlu-pad vs. Patching. BCVA significantly differed between Occlu-pad and Patching at 6, 9, and 12 months (^∗^p=0.02, ^∗^p=0.02, ^∗^p=0.03; ANOVA).

**Figure 3:**
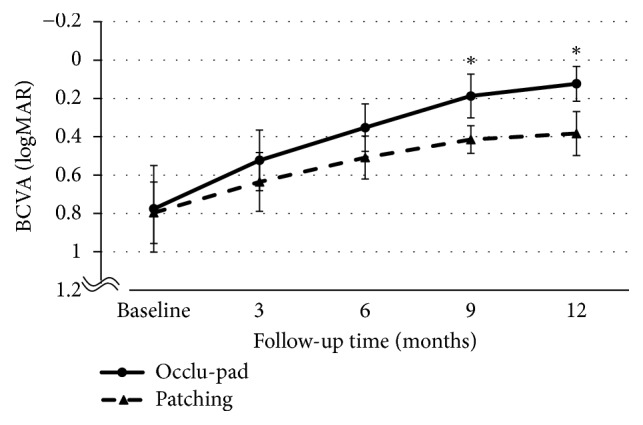
Best-corrected visual acuity (BCVA) for strabismic amblyopia patients, from baseline to 3, 6, 9, and 12 months. Occlu-pad vs. Patching. BCVA significantly differed between Occlu-pad and Patching at 9 and 12 months (p=0.03, p=0.03; ANOVA).

**Figure 4:**
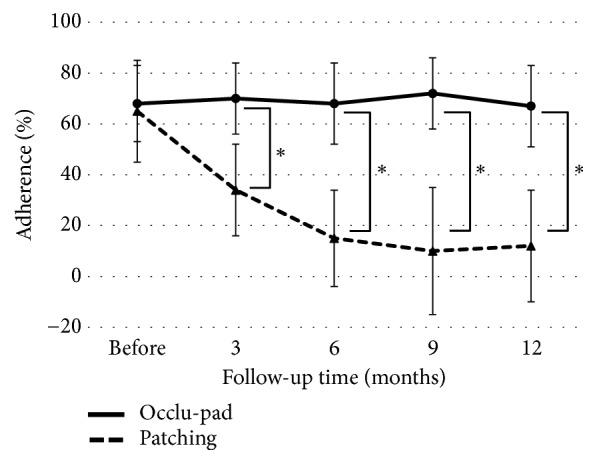
Changes in adherence over time. After 6 months, adherence fell 20% in the Patching group. All ^∗^P values were < 0.001 (Mann-Whitney U test).

The Occlu-pad and Patching groups both showed improvement. For anisometropic amblyopia, visual acuities of the Occlu-pad/Patching groups before treatment were 0.78 ± 0.35/0.80 ± 0.36 logMAR; after 6 months, the visual acuities were 0.09 ± 0.25/0.33 ± 0.25 (p < 0.05). Statistically significant differences were observed in visual acuity improvement for both Occlu-pad and Patching groups from the 6th month onward (Figure 2). For strabismic amblyopia, the visual acuities of the Occlu-pad/Patching groups before treatment were 0.77 ± 0.39 / 0.79 ± 0.46 logMAR; after 9 months, the visual acuities were 0.18 ± 0.22/0.41 ± 0.14 (p < 0.05) (Figure 3). Moreover, Occlu-pad / Patching groups exhibited 70% / 34% adherence after 3 months of training; significant differences in adherence were observed after 3 months and continued throughout the study (Figure 4). The adherence of the Occlu-pad group remained at 68% to 72% for 6 months.

### 3.2. Discussion

We compared visual acuity improvement in patients with amblyopia between Occlu-pad and Patching treatment approaches. We found statistically significant changes in visual acuity after 6 months of treatment in both groups. Regarding training adherence, the Occlu-pad group showed significantly higher adherence than the Patching group after 3 months of training. The adherence of the Occlu-pad group remained at 68% to 72% for 6 months.

For amblyopia training with a blocking closure such as an eye zone, maintaining adherence to the prescribed training time is a primary problem [10–12]. Previous reports showed that the adherence to training time was approximately 50% [7] and dropped to 20% [4] after 3 months. In the present study, Patching adherence decreased gradually from the first month and reached 34% by the third month, consistent with the previous report. In contrast, with amblyopia training using Occlu-pad, it was possible to maintain adherence to prescribed training time for 6 months. Thus, by changing the training method, we were able to improve adherence. Although there is variation based on the age and unique situation of each patient, it is often difficult for a child to continue the occlusion-based closure method with eye zones. In clinical practice, Patching is sufficient, but care should be taken when no remarkable treatment effect is observed over a long period of time [12]. There is a limit to the self-reporting of training time provided by parents. Evaluation of adherence with evidence of the therapeutic effect is important. Because the Occlu-pad can automatically record training time inside the device, accurate training time and treatment effect can be examined. In addition, despite its shorter training time (compared with Patching), training by Occlu-pad resulted in a greater training effect. Conventional Patching is a method to train amblyopic eyes by occlusion. Conversely, training with Occlu-pad involves open eyes. With respect to amblyopia training with open eyes [6], there have been reports [13] that the activity of the occipital lobe visual cortex on both sides (change in oxygenated hemoglobin kinetics) is significantly greater than that observed with single-eye occlusion. Thus, amblyopia training with open eyes may provide better results [14]. Moreover, Patching could not be implemented as instructed, but the possibility that it was not actually done cannot be denied.

## 4. Conclusions

This study showed that the Occlu-pad amblyopia training method was able to maintain high motivation for amblyopia training. As a new method of amblyopia training, children and their families can listen to “Patch or play?” Thus, training methods can be proposed according to patients' lifestyles and might be useful in amblyopia training. Our results suggest that amblyopia training by Occlu-pad may be helpful as a new amblyopia treatment.

## Figures and Tables

**Table 1:** Patient characteristics.

	Occlu-pad n=72 (%)	Patching n=66 (%)
Male	36 (50)	36 (55)
Female	36 (50)	30 (45)

Age at enrollment		
3 to < 5 years	29 (40)	24 (36)
5 to 7 years	25 (34)	20 (30)
7 to 9 years	18 (25)	22 (33)
Mean ± SD	5.5 ± 1.7	5.0 ± 1.5

Type of amblyopia		
Anisometropia	35 (48)	35 (53)
Strabismus	37 (51)	31 (46)

Maximum magnitude of tropia		
Deviation at distance measured by SPCT,Δ		
Orthotropic	42(58)	34(52)
1-9	12(17)	14(21)
10>=	18(25)	18(27)

Maximum magnitude of tropia		
Deviation at near measured by SPCT,Δ		
Orthotropic	42(58)	36(55)
1-9	8(11)	18(27)
10>=	22(31)	12(18)

Distance visual acuity in amblyopic eye	(LogMAR)	
1.3 to 1.0	22 (30)	20 (30)
0.8 to 0.4	30 (41)	26 (39)
0.3 to 0.15	20 (27)	20 (30)
Mean ± SD	4.5 ± 1.7	4.2 ± 1.2

Distance visual acuity in fellow eye	(LogMAR)	
0.1 to 0.0	4 (5)	6 (9)
≥ -0.1	68 (94)	60 (90)
Mean ± SD	-0.05 ± 0.1	-0.05 ± 0.1

Refractive error in amblyopic eye (SE)		
0 to < +4.00D	15 (20)	12 (18)
+4.00D to < +7.00D	41 (56)	35 (53)
≥ +7.00D	16 (22)	19 (28)
Mean ± SD (Diopter)	5.5 ± 2.4	5.8 ± 1.6

Refractive error in amblyopic eye	(Astigmatism)	
0.00D to <0.50D	21(29)	21(32)
0.50D to <1.00D	15(21)	10(15)
1.00D to <1.50D	16(22)	18(27)
≥1.50	20(28)	17(26)
Mean ± SD(Diopter)	1.0±0.9	1.2±1.0

Refractive error in fellow eye (SE)		
0 to < +4.00D	55(76)	53(80)
+4.00D to < +7.00D	11(15)	7(11)
≥ +7.00D	6(8)	6(9)
Mean(SD)D	2.8±3.0	3.1±3.9

Prior amblyopia treatment		
None	60(83)	66(91)
Patching	9(13)	3(5)
Atropine	0(0)	2(3)
Patching and atropine	3(4)	1(1)

## Data Availability

The data are not available for public access because of patient privacy concerns but are available from the corresponding author on reasonable request.

## References

[1] Holmes J. M., Clarke M. P. (2006). Amblyopia. *The Lancet*.

[2] Van Leeuwen R., Eijkemans M. J. C., Vingerling J. R., Hofman A., De Jong P. T. V. M., Simonsz H. J. (2007). Risk of bilateral visual impairment in individuals with amblyopia: the Rotterdam study. *British Journal of Ophthalmology*.

[3] Pediatric Eye Disease Investigator Group (2002). A randomized trial of atropine vs. patching for treatment of moderate amblyopia in children. *Arch Ophthalmol*.

[4] Wallace M. P., Stewart C. E., Moseley M. J., Stephens D. A., Fielder A. R. (2013). Compliance with occlusion therapy for childhood amblyopia. *Investigative Ophthalmology & Visual Science*.

[5] Repka M. X., Beck R. W., Holmes J. M. (2003). A randomized trial of patching regimens for treatment of moderate amblyopia in children. *JAMA Ophtalmology*.

[6] Kelly K. R., Jost R. M., Dao L., Beauchamp C. L., Leffler J. N., Birch E. E. (2016). Binocular ipad game vs patching for treatment of amblyopia in children: a randomized clinical trial. *JAMA Ophthalmology*.

[7] Stewart C. E., Stephens D. A., Fielder A. R., Moseley M. J. (2007). Objectively monitored patching regimens for treatment of amblyopia: Randomised trial. *British Medical Journal*.

[8] Tjiam A. M., Holtslag G., Vukovic E. (2012). An educational cartoon accelerates amblyopia therapy and improves compliance, especially among children of immigrants. *Ophthalmology*.

[9] Handa T., Ishikawa H., Shoji N. (2015). Modified iPad for treatment of amblyopia: A preliminary study. *Journal of American Association for Pediatric Ophthalmology and Strabismus*.

[10] Dean S. E., Povey R. C., Reeves J. (2016). Assessing interventions to increase compliance to patching treatment in children with amblyopia: A systematic review and meta-analysis. *British Journal of Ophthalmology*.

[11] Al-Yahya A., Al-Odan K., Allam K., Al-Onazi B., Mousa A., A. Al-Saleh A. (2012). Compliance to patching in the treatment of amblyopia. *Saudi Journal of Ophthalmology*.

[12] Rajavi Z., Mokhtari S., Sabbaghi H., Yaseri M. (2015). Long-term visual outcome of congenital cataract at a Tertiary Referral Center from 2004 to 2014. *Journal of Current Ophthalmology*.

[13] Iwata Y., Handa T., Ishikawa H., Shoji N., Shimizu K. (2016). Efficacy of an amblyopia treatment program with both eyes open: A functional near- infrared spectroscopy study. *American Orthoptic Journal*.

[14] Li J., Thompson B., Deng D., Chan L. Y. L., Yu M., Hess R. F. (2013). Dichoptic training enables the adult amblyopic brain to learn. *Current Biology*.

[15] Wikipedia Occlu-pad. https://ja.wikipedia.org/wiki/%E3%82%AA%E3%82%AF%E3%83%AB%E3%83%91%E3%83%83%E3%83%89.

